# Maintaining safety and efficacy for 3D printing in medicine

**DOI:** 10.1186/s41205-016-0009-5

**Published:** 2017-01-26

**Authors:** Andy Christensen, Frank J. Rybicki

**Affiliations:** 1 SOMADEN LLC, Littleton, CO USA; 2 Department of Radiology, University of Ottawa Faculty of Medicine, 501 Smyth Road, Ottawa, ON K1H 8L6 Canada; 3 The Ottawa Hospital Research Institute, Ottawa, ON Canada

**Keywords:** 3D printing, Radiology, Regulatory, Anatomical model, Modified anatomical model, Virtual surgical planning, Medical model, Additive manufacturing, Medical devices

## Abstract

**Background:**

The increased and accelerating utilization of 3D printing in medicine opens up questions regarding safety and efficacy in the use of medical models. The authors recognize an important shift towards point-of-care manufacturing for medical models in a hospital environment. This change, and the role of the radiologist as a central facilitator of these services, opens discussion about topics ranging from clinical uses to patient safety to regulatory implications.

**Results:**

This project first defines three groups of patients for whom 3D printing positively impacts patient care. The steps needed for each group are described.

**Conclusions:**

We provide our opinions regarding the regulatory role that we feel is most appropriate, balancing safety and efficacy with the autonomy of individuals in the field to make the greatest positive impact on healthcare.

## Introduction

3D printing from volumetric medical images has entered a phase of steep growth. Although this growth has provided an appearance that the medical modeling application is nascent, 3D printing for surgical planning and the use of 3D printing to develop tools to enhance medical procedures has a rich history in both private practice and academic medicine [1–15]. Medical device manufacturers and individual industry leaders have worked with the U.S. Food and Drug Administration (FDA) to empower healthcare providers with innovative, personalized devices that are safe and effective, while academicians have devised and tested new interventions that would not be possible without 3D printing [16–20]. For these procedures, the Center for Devices and Radiological Health (CDRH) at the Food and Drug Administration (FDA) has reviewed and cleared 3D printed medical devices for more than 10 years [21]. An important shift is happening towards point-of-care manufacturing for medical models in a hospital environment. This change, and the role of the radiologist as a central facilitator of these services, opens discussion about topics ranging from clinical uses to patient safety to regulatory implications.

Three important regulatory milestones should be acknowledged. First, the October 2014 FDA Public Workshop [22], “Additive Manufacturing of Medical Devices: An Interactive Discussion on the Technical Considerations of 3D Printing”, held by an FDA Working Group that detailed best practices for 3D printing quality and safety as well as an assessment of medical devices, followed by discussion of topics ranging from bioprinting to pharmacoprinting to metals implant printing. Approximately 500 people attended this two-day workshop with another few hundred watching online from around the world. The second milestone is the FDA “leapfrog” Draft Technical Guidance Document, “Technical Considerations for Additive Manufactured Devices” [23], released for public comment on May 10, 2016. The third milestone was the publication “Additively Manufactured Medical Products – The FDA Perspective” [21], that further explored the topic, including 3D printing of patient-specific anatomy and the 3D printers used for such tasks.

This document provides the authors’ collective opinion, and frames the quality and safety challenges that we perceive have emerged from state of the art medical 3D printing. We engage the FDA and the overall community to achieve needed clarity and specifics regarding the potential benefits achievable through standardization. We are determined to foster a conversation that ensures safety and efficacy for a large number of medical models, and to group these models with respect to risk, which may correlate to the regulatory classification associated with specific applications. Increasing complexity comes from using digital data from patient anatomy to further plan surgery and to provide 3D printed instruments, templates, and models to facilitate surgical intervention.

For the purposes of this article the common applications today for 3D printing in medicine will be delineated by their intended use, which may also roughly correlate to how the FDA views these applications. The three application groups to frame this discussion are:

- Group I. Anatomical Models. A model representing as-scanned anatomy intended for visualization, surgical planning, education, informed consent and reference during surgery. Intended use: 3D reference of anatomy to aid surgical planning
- Group II. Modified Anatomical Models. A modified model of anatomy, simple surgical planning performed digitally to further enhance the model, significantly modified models. Intended use: enhanced surgical planning and guidance
- Group III. Virtual Surgical Planning with Templates. Complex surgical planning done digitally, 3D printed templates/models/guides produced which are intended to guide the digital plan on the patient in the operating room. Intended use: to augment the surgical procedure with specific pre-planned steps which are carried out in surgery using 3D printed guides or templates.

**Fig. 1:**
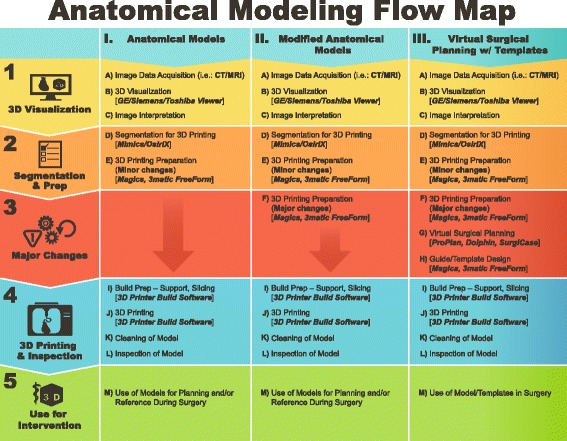
Anatomical Modeling Flow Map

To further help clarify the types of devices that exist and regulatory ramifications for each of the three groups, (Fig. 1) details those common steps A-M to produce a 3D printed model from medical imaging data. Typically, the DICOM standardized format is the presumed starting point for medical 3D printing as described in this document.

A. Image Data Acquisition – Volumetric medical imaging studies, for example, Computed Tomography (CT) is used preferentially for bone or hard tissues, and Magnetic Resonance Imaging (MRI) may be used for cardiovascular printing and for printing soft tissues. DICOM images are the output of this first step.
B. 3D Visualization – For the vast majority, or all, patients for whom 3D printing is desired, the image interpretation (clinical reporting) will include one or more of the following tools: multiplanar reformatting, maximum intensity projections, and volume rendering. Collectively, these DICOM data manipulations are termed “3D visualization” [24]. Most patients who benefit from 3D visualization will not require, or undergo, 3D printing. For example, a patient who presents to Emergency Medicine with right upper quadrant pain, suspected cholecystitis, and a normal sonography may necessitate CT that identifies a retrocecal appendix. Reformatted images will assist surgical planning, but 3D printing is not indicated, nor would it be considered temporally reasonable in a patient who would benefit from timely surgery. 3D visualization may be performed using software associated with the imaging hardware, or software from an alternate vendor.
C. Image Interpretation – The medical images are typically interpreted before the decision to use 3D printing to clinically improve the individual patient’s outcome. Of note, 3D printed models can in theory reveal additional data in patient anatomy to enhance diagnoses, and in these situations an addendum to the medical imaging report can be rendered.
D. Segmentation for 3D Printing – This step begins with DICOM images from the medical imaging study and allows for segmentation to transform them into an unaltered surface-based 3D model that can subsequently be used for 3D printing.
E. 3D Printing Preparation: Minor Changes – Simple changes are made to make the model more “printable” or to illustrate anatomical structures by labeling or coloring. “Minor” versus “major” changes are an important distinction and will be discussed in detail.
F. 3D Printing Preparation: Major Changes – Modifications to the model that facilitate intervention planning. These will be detailed further, but include electronically resecting and reconstructing structures, providing graft/implant templates, or geometric mirroring to ascertain symmetry.
G. Virtual Surgical Planning – More detailed planning of the surgical intervention in digital space, whereby the digital plan will be transferred to the patient by way of 3D printed templates, guides, or models.
H. Guide/Template Design – When virtual surgical planning is performed, the plan is transferred to the patient with personalized instruments in the form of templates, guides or models which need to be designed using the output of Step G.
I. Build-Preparation, Support, Slicing – Files are processed further for the specific 3D printer to be used, taking into account the material and additions such as support structures and layer thickness. The output file from this step will be input into the 3D printer.
J. 3D Printing – Generating the output model covers a wide range of techniques. Categorization of differences includes how the model is constructed, the energy source, type of materials, and other factors [25].
K. Cleaning of Model – Depending on the 3D printing technique, the model will need to be cleaned of residual manufacturing materials and/or substances required to clean the model.
L. Inspection of Model – Accuracy of the model is paramount; this step can use qualitative and/or quantitative measures to confirm that the 3D printed model matches the desired input data.
M. Model Used by Physician – Typical uses for anatomical models span a large gamut from patient consent, to anatomic and procedural training, and from determining the surgical plan to actually guiding the procedure itself.

### Medical Imaging: Steps A-C

Steps A-C follow current medical imaging paradigms for image acquisition and interpretation. Hardware and software that are used to generate medical images and post-process them for interpretation are evaluated by the FDA and cleared/approved for their intended use.

### Segmentation & preparation for 3D printing: Steps D-E

Step D, in which DICOM images are segmented to create STL files, should use software that is FDA cleared for this purpose [21]. The rationale for why FDA-cleared software should be used for creation of the STL file is two-fold. First, a significant fraction, if not the large majority, of this segmentation step largely parallels the second step, 3D Visualization, from which the volumetric data is post-processed to generate, for example, multiplanar reformatted images, maximum intensity projections, and 3D volume renderings. Just as 3D visualization software tools require FDA clearance, so should software used for this step, based on perceived risk and safety. The second part of the rationale is that the largest potential error, that is differences between the anatomy captured within the imaging step and the 3D printed model, will arise at this step. These errors can be attributed to either humans or software. The latter should be minimized so as to optimize patient safety. Minor changes to the data for better 3D printing or to highlight an area or for support are done in preparation for 3D printing.

### Major changes to the data: Steps F-H

While there is alteration of the medical imaging data in the prior steps, Steps F-H differ because anatomy is altered to facilitate a given, patient-specific intervention. Modified Anatomical Models have been augmented in some way for a patient-matched therapy. Virtual surgical planning further extends these concepts, actually planning the surgical intervention which will be carried out using personalized instruments, templates or models which are designed following the completion of the surgical planning.

### 3D printing: Steps I-L

The steps involved in any of the numerous 3D printing techniques [25] cover preparation of the files before printing, the 3D printing process itself, and model cleanup following production and inspection of the final model. Inspection will be dependent on the application and need for accuracy, and could include qualitative and quantitative measures. Some applications will test the limits of the process and require the user to use a robust validation to prove reliability [23, 26].

### Device use: Step M

Models may be used by surgeons and many other types of interventionists to prepare for a patient specific intervention. Educating junior professionals and obtaining informed consent are also often discussed. Anatomical models and 3D printing-centric therapies have been shown to reduce operative time for certain procedures [27] and increase procedural accuracy in others [20].

### Group I. Anatomical models

#### Description

**Fig. 2:**
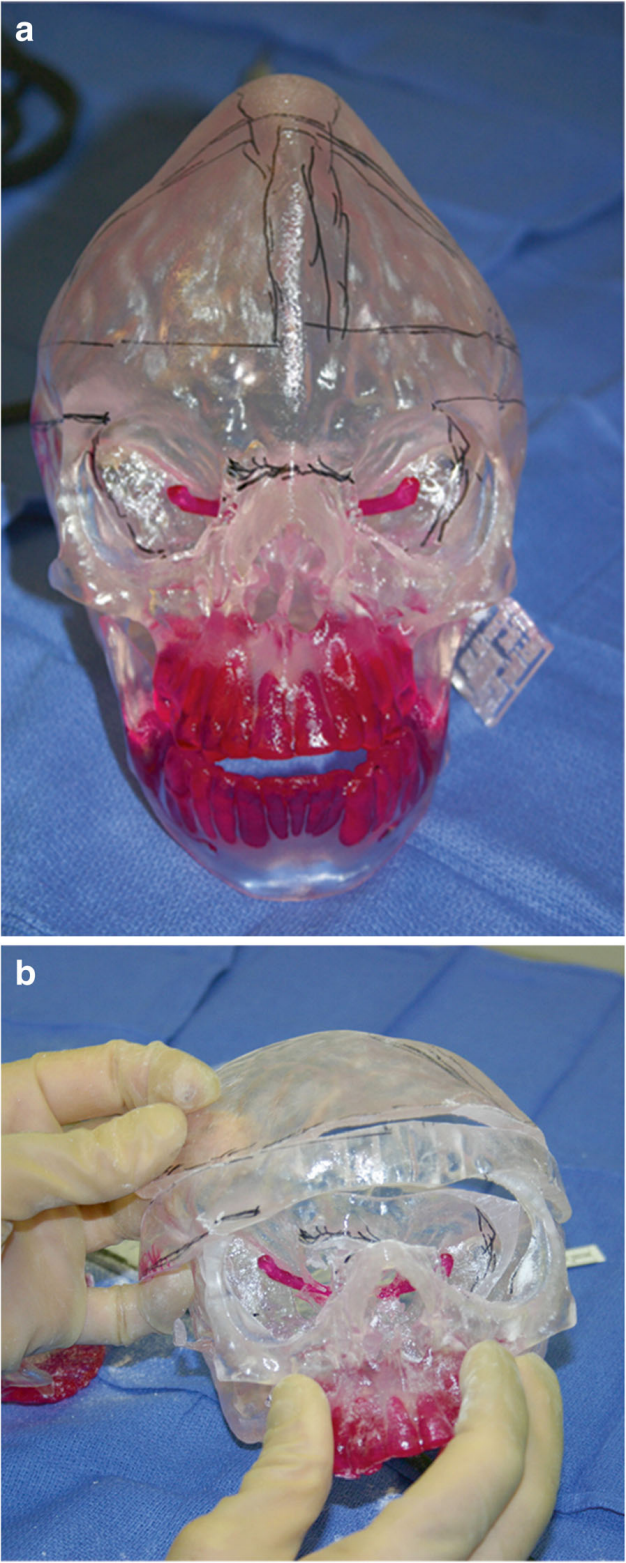
Group I, Anatomical Models. The 3D printed model replicates the anatomy captured by medical imaging. Patient with Crouzon syndrome, note the characteristic calvarial deformity. **a**, the optic nerve is colored *red* for planning intervention. **b**, dissected model, after practice intervention. In this case, combined neurosurgery and craniofacial surgery included removing and reshaping portions of the cranial bone. Photos courtesy Dr. Kenneth E. Salyer, Dallas, TX

These are models of anatomy to be used for education, surgical planning, patient consent, and reference during surgery. Pathology may be highlighted but the anatomic data captured by volumetric imaging is not altered (Fig. 2).

#### Applicable Steps: A-E, I-M

Typically derived from CT or MRI, Group I 3D models are designed to depict the anatomic information contained in the medical images, without alteration, and are generally used for surgical planning. Examples include a newborn with double outlet right ventricle [28] or craniomaxillofacial models where tissues are not altered, for example to plan complex procedures including congenital deformity corrections and secondary reconstruction following trauma [2, 27, 29]. Orthopedic [30] and cardiovascular [19] applications include 3D printed models for visualization and physical, hands-on simulation. The common denominator for Group I models is that 3D printing extends current 3D visualization and the model does not differ from the patient’s anatomy. Steps A-E, including “3D Printing Preparation” should use systems (software and some hardware) that are cleared by the FDA for their intended use. This includes not only the imaging hardware/software, but also the software used to segment the medical images from their original 2D state into a 3D dataset usable for 3D printing (i.e., an STL or 3MF file [31]). Regarding the steps “Build Prep – Support, Slicing” to the end of the pathway when models are used by healthcare professionals, work should not be under the auspices of the FDA and do not contain what the FDA considers to be Medical Devices [32]. Instead, these should be managed by the entity that manufactures the model (e.g., industry or hospital) and treated as being used by doctors and other healthcare workers as a tool in the practice of medicine.

In this scenario, Step E “3D Printing Preparation – Minor Changes” does not make significant changes to the anatomy, but instead refines the STL file so that it can be printed. Since the anatomy of the printed part has been finalized in Step D, these minor modifications are grouped with the later steps in the workflow of generating the model after the completion of the design. Minor modifications have the intent of not modifying the original anatomy, but rather highlighting an area, labeling the model, or adding material to allow for better 3D printing production. The following are examples of minor modifications of the models:

1. Filling holes in anatomy introduced by imaging artifact
2. Smoothing anatomy in anatomical areas where imaging artifact has been introduced
3. Adding structural supports to keep anatomy in proper relation to other anatomy
4. Adding material as a “wall” around a contrast-enhanced object (such as a vessel lumen), commonly used for heart or vascular modeling
5. Removing known imaging artifact
6. Labeling the model
7. Cutting the model into parts for better visualization or 3d printability
8. Adding magnets to allow for better functionality of cut models
9. Adding color to delineate or highlight anatomical structures
10. Grouping separate anatomical structures into a single model file

We recognize that software packages may be designed to do these preparation steps as well as more major modifications spelled out in Step F. However, patients who require substantial modifications to the anatomy captured in the medical images should be considered in Group II: Modified Anatomical Models. While software used for Step E may have FDA clearance, we believe it is not required for this intended use. Moreover, FDA clearance of additional devices is not required for the steps following Step E for Group I models. These steps involve the fabrication of the 3D printed model and subsequently the inspection and utilization of the model for patient care. The 3D printed model which is the output of this path is not considered a Medical Device by the FDA [21].

#### Intended use

Intended for use as reference physical model of anatomy which is the result of a volumetric medical imaging study combined with further image processing and 3D printing.

#### US FDA regulatory classification

The end product 3D printed models are not considered Medical Devices by the FDA [21]. Steps up to and including “D. Segmentation” do require use of a properly cleared/approved Medical Device.

#### Regulatory explanation

The end product 3D printed models fall outside of the FDA’s purview [21]. Steps up to and including “D. Segmentation” always require use of a properly cleared/approved Medical Device.

#### Example devices

Models include the following as-scanned anatomy from an in-hospital 3D printing laboratory [33, 34] for surgical planning: hard tissue models of the bone showing congenital deformity, trauma or acquired condition for reconstructive surgery; soft tissue vascular models to show challenging anatomy for intervention, such as an abdominal aortic aneurysm under consideration for open versus less invasive repair; renal models used to plan a partial versus total nephrectomy.

#### FDA listings/clearances

Not applicable; the FDA does not consider the end-product anatomical model to be a Medical Device. The model is considered a hard copy output, akin to printing medical images on film [21].

### Group II. Modified anatomical models

#### Description

**Fig. 3:**
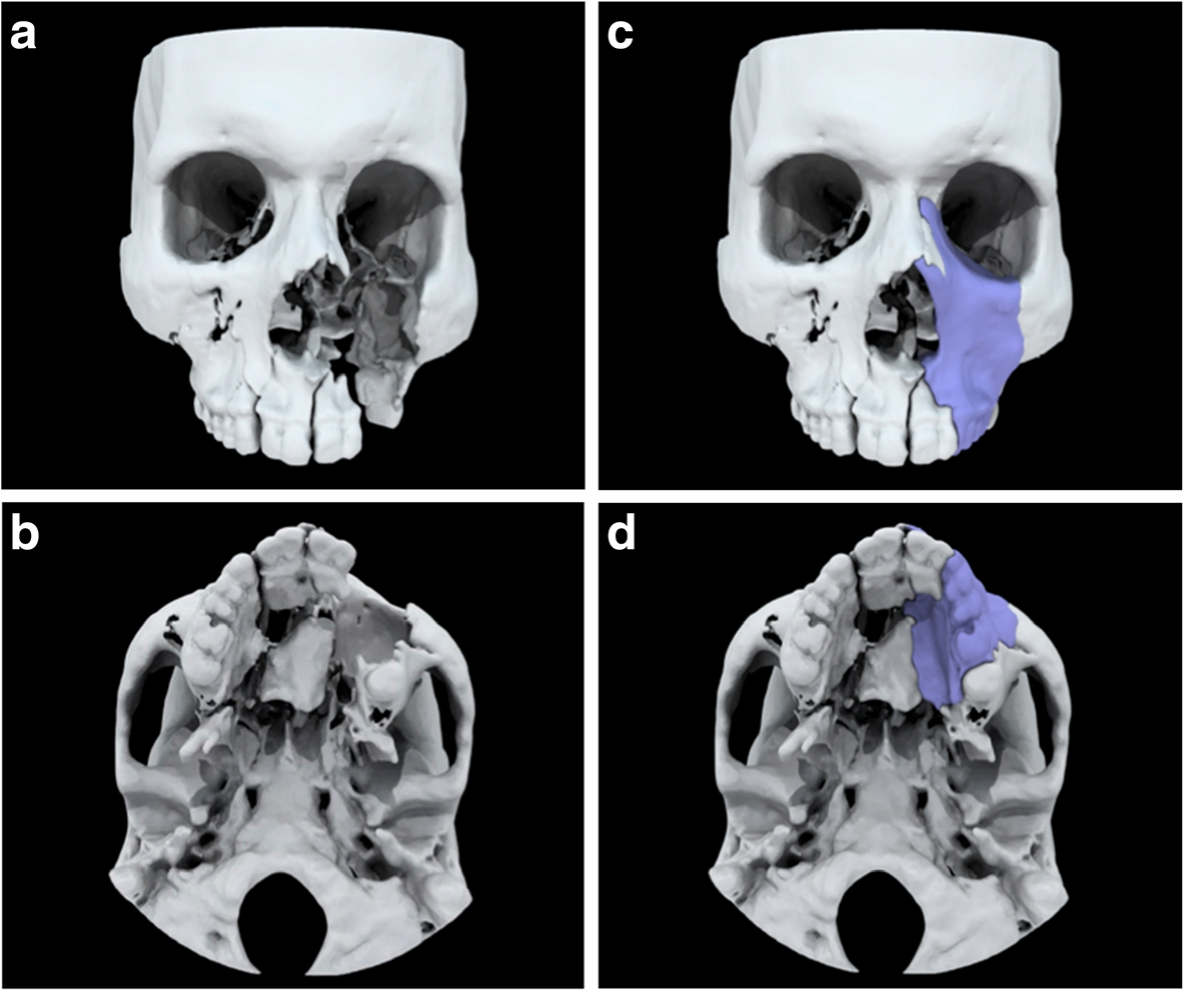
Group II, Modified Anatomical Models. Example modifications to the anatomy rendered from medical imaging. **a** and **b**, Patient after trauma to the maxilla has a large osseous defect, including teeth. **c** and **d**, Model demonstrating design of a suitable graft to facilitate surgical reconstruction. Missing bone is designed using the contralateral anatomy and output as a separate file for 3D printing a template (*purple*)

Models of anatomy whereby the anatomy has been significantly altered from the “as-scanned” state to further facilitate surgical planning or other intervention. Examples include mirroring anatomy, resecting or reconstructing anatomy, or designing grafts (Fig. 3).

#### Applicable Steps: A-F, I-M

3D models that fall into this category have the same steps A-D as the non-modified anatomical models described above. Typically, volumetric imaging is acquired and the same steps up to segmentation are performed, with the hardware and software all requiring clearance for their intended uses from the FDA. The main difference between models in Group I and those in Group II is patients who are modeled in Group II have both F (major changes) plus E (minor changes), whereas patients who are modeled in Group I undergo minor changes alone. Step F, unique to Group II modeling, includes further modifications to the file that are made for a patient-specific intervention. The following are examples of major modifications:

1. Removal of segmented anatomy such as a tumor, in order to visualize the size of the defect before reconstruction
2. Mirroring of the dataset to provide a mirror-image model in order to ascertain degree of symmetry or asymmetry
3. Mirroring and “perfecting” of the dataset to provide a model which appears to be “perfect” (a unilateral defect has now been erased by combining half of a mirror-image model with the half of the unaffected original patient model),
4. Digital placement of a “graft” of either alloplastic or autogenous material into a defect and including this on the resultant model
5. Visualizing, sizing, and simulating intervention using another medical device digitally and subtracting said device, its shadow, screw holes/fixation points, etc. from the original model, leaving an imprint/pattern/holes of some type on the model
6. Visualizing, sizing and simulating intervention using another medical device and altering the model in some way that includes this device
7. Designing a graft of alloplastic or autogenous material and printing out the model with an indication of this graft or a printout of the new graft itself. An example includes a patient with a cranioplasty defect and filling the defect with a perfectly-fitting implant template which will be used to guide harvest of autogenous material or shaping/manufacturing of alloplastic material.

When one or more of these Step F, or major modifications, is made, or a comparable modification that changes the patient’s anatomy in planning for a specific intervention, the software used to make that modification should be FDA cleared for this intended use, and the printed model should also be considered a Medical Device. Subsequently, the device itself will require 3D printing. When 3D printing a medical device, the hardware (i.e., the 3D printer) and the software used to drive the hardware should be FDA cleared for these intended uses. The rationale is that a large number of 3D printing technologies, with varying levels of performance and capabilities, could be otherwise used to print medical devices that could, in turn, be used for more intensive guidance of treatment.

#### Intended use

Intended for use as a reference anatomical model by a surgeon/interventionist to plan a surgical intervention. Modifications made to the anatomy are intended for patient-specific intervention planning and/or guidance during surgery.

#### US FDA regulatory classification

These models are considered Medical Devices [23]. Some of these are Class I Exempt Medical Devices (i.e.,: FDA Product Code HWT – Template for Clinical Use) while some of these (e.g., cranioplasty templates and others) would be up-classified to the level of the implant systems they support, typically Class II.

#### Regulatory explanations

*For a commercial company* to legally market and sell a product, the manufacturer must be registered with the FDA, have an FDA-compliant quality system and list the product with the FDA. FDA pre-market approval (i.e.,: 510(k)) would not be required for a Class I Exempt device, but would likely be required for any Class II devices. The entity is subject to upholding the FDA’s Quality Systems Regulations (QSR) and can be inspected by the FDA at their leisure.

*For a hospital entity*, the hospital should seek to use a cleared Medical Device “system” for production of these types of models in a hospital setting.

### Example FDA listings/clearances

- Materialise HeartPrint – [35]
- 3D Systems ClearView Anatomical Model – [36]
- Anatomical models sold by numerous companies such as 3D Systems [37], Materialise [38], DePuy Synthes [39] and Stryker [40].

### Group III. Virtual surgical planning with templates

#### Description

**Fig. 4:**
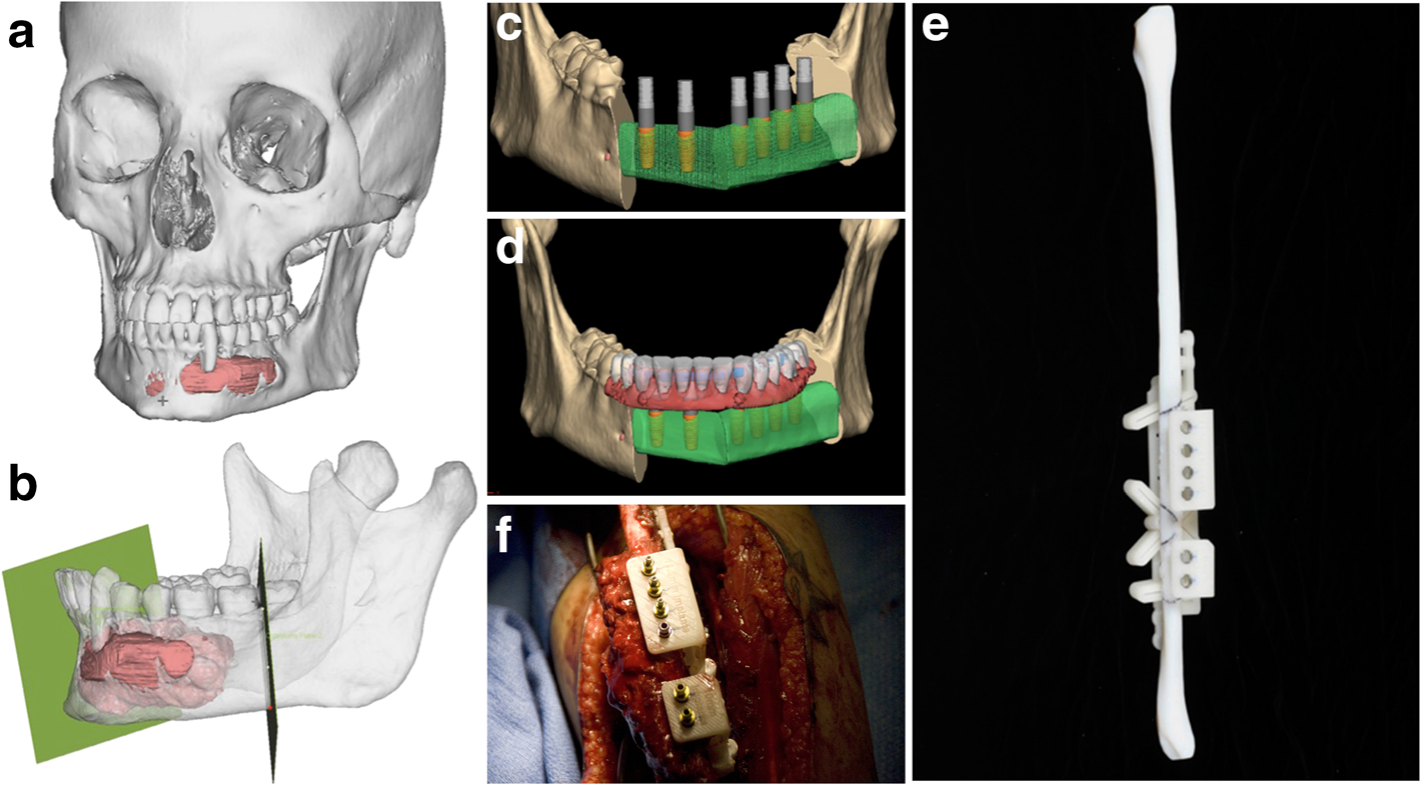
Group III, Virtual Surgical Planning with Templates. **a**, 3D printed model of patient with aggressive lesion of the mandible, colored in *red*. Radiology interpretation and the 3D model helped decide on a treatment plan including full thickness bony resection. The surgical reconstruction plan was designed for a free fibula graft, that is, a vascularized fibula graft containing bone and soft tissue. **b**, Cutting paths for the bone resection (*green*). **c**, virtual placement of the fibula graft (*green* bone segments); *grey and red* cylinders represent dental implants to be placed at the time of surgery. **d**, Virtual placement of a denture supported on this framework, all to be planned and accomplished a single-stage procedure. **e** and **f**, 3D printed templates to enable precise osteotomies of the fibula and customized dental implant positions were produced with stereolithography and used in surgery, enabling what otherwise would not be possible within the scope and time of a single intervention. Photos courtesy David L. Hirsch, DDS, MD, Chief, Oral and Maxillofacial Surgery, Lennox Hill Hospital, NY, New York

Virtual Surgical Planning is a digital process that typically begins with a volumetric medical imaging study. Proven applications include bone reconstructive surgery such as repair of congenital defect, or after trauma to the craniomaxillofacial region [16–18] or other orthopedic applications. Once surgery is planned digitally, templates and guides are then designed to transfer the surgical plan from the computer to the patient (Fig. 4). Models of anatomy may be present but more important are the 3D printed templates/guides/models that enable transfer of a digital surgical plan to patient care. Another common use of virtual surgical planning (Group III) is for templating total joint arthroplasty whereby the surgical simulation helps choose size/model of the implant and the template properly positions the implant or prepares the patient’s bone structure, taking into account the patient’s unique anatomy and physiology.

#### Applicable Steps: A-M

##### Intended use

For those virtual surgical planning devices with a software component, the intended use may also include the following: intended for use as a software interface and image segmentation system for the transfer of CT or MRI data. The input data file is processed and the result is an output data file that may then be used as input data for a 3D printer that produces anatomical models, templates, and surgical guides. Also intended as a pre-operative software tool for simulating/evaluating surgical treatment options, or templating for total joint arthroplasty. FDA’s product code PBF, “orthopedic surgical planning and instrument guides” [41] combines both the 3D printed output guides/templates along with the surgical planning software. It is expected that this will happen more frequently as further applications arise for personalized surgical guidance.

##### US FDA regulatory classification

These products are primarily considered Class II Medical Devices subject to premarket clearance requirements, most times requiring a 510(k). Class III Medical Device(s) requiring a Premarket Approval (PMA) have also been approved in this area for use in templating Class III implant systems.

#### Regulatory explanations

*For a commercial company* to legally market and sell the product, the manufacturer must a) have clearance (510(k)) or approval (PMA) for the product, b) be registered with the FDA, c) have an FDA-compliant quality system, and d) list the product with the FDA.

*For a hospital entity*, the hospital should seek to use an FDA-cleared Medical Device “system” for production of these types of models/templates/guides/surgical plans.

### Example FDA listings/clearances

Class II Clearances:

- Materialise ProPlan/SurgiCase – 510(k) K111641 [42]
- Zimmer Biomet Signature – 510(k) K133162 [43]
- 3D Systems VSP– 510(k) K120956 [44]
- DePuy Synthes TruMatch– 510(k) K110397 [45]

## Review

The number of medical 3D printing applications continues to grow, as does the number of users dedicated to improving patient care pathways. The authors recognize potential divergent pathways for patient safety and the efficacy of medical models, and they provide recommendations for dialogue on best methods to integrate 3D printing to medical practice. 3D printing applications are separated into three groups: Anatomical models, Modified anatomical models, and Virtual surgical planning with templates. These groups are explicitly defined with recommendations on optimizing safety and efficacy for each. Professionals engaged in 3D printing must maintain a high level of training and competence, and we recommend the use of FDA cleared software and hardware when appropriate.

## Discussion

### Hospitals making parts versus companies selling medical devices

In the United States, the FDA’s Center for Devices and Radiological Health (CDRH) is responsible for regulating firms who manufacture, repackage, relabel, and/or import medical devices sold in the United States [32, 46, 47]. In practical application the FDA regulates those who market and sell devices to the U.S. market; these are the Original Equipment Manufacturers (OEMs) of medical devices, for example Johnson & Johnson, Stryker, and Medtronic. The FDA does not typically regulate the users of these devices, although there are likely exceptions to this rule. While hospitals are not typically under the FDA’s purview, in the U.S. many are often accredited by the independent Joint Commission (formerly the Joint Commission on Accreditation of Healthcare Organizations – JCAHO [47]). The Joint Commission audits and provides accreditation to hospitals providing quality medical care.

Other countries may have similar or different views on what does or does not constitute a medical device, from a regulatory standpoint. We suggest that users outside of the U.S. contact their local authority to ascertain who regulates medical devices and to determine what is regionally appropriate.

### Areas of potential concern

We recognize that, to date, physicians who have been utilizing 3D printing for all three groups defined above have held very high standards, and many have benefited from FDA oversight. Several reasons exist today which have created circumstances where a future safety risk is real. These include:

- Freeware software of unknown quality/pedigree being used to process patient images.
- Freeware software of unknown quality/pedigree being used to plan, simulate, or perform “virtual surgery”
- 3D printers are now available almost everywhere, from a $100 model to a $1,000,000 model. Lower end models may not be suited towards the fabrication of quality medical devices; however, users can adopt technologies because they are inexpensive.
- Unknown expertise of operators using non-cleared software devices is of concern, particularly if models are intended to guide a medical intervention.

Relating specifically to software, the FDA has given guidance in the past years for Commercial off the Shelf Software (COTS) and Software of Unknown Provenance (SOUP), relating to software that may be used as part of a medical device but with unknown development path and/or safety record [26, 48]. The issue is not only within the COTS/SOUP software, but also when it serves a key role in a process the issue is with the rest of the software “system”, and potentially negative/unknown effects on the whole of the process.

### The call for standardization and guidelines

We recognize that, even with clarity regarding the material presented above, several questions remain. These too are important to tackle given the exponential growth in the field. The first question relates to a possible different set of recommendation for individuals working inside a clinical facility (e.g., hospital-based 3D printing) versus obtaining models from an outside vendor. We believe that there should be no distinction. The rationale is that both sets of professionals should have the same standards, and provide the same profile of safety and efficacy. As utilization of 3D printing in medicine increases from both groups, rigorous guidelines are needed to ensure that the field progresses with a “patient-first” interest as the top priority.

Today, medical 3D printing has largely focused on niche applications, and members of the relatively small community have held very high quality standards. There are published metrics for model reproducibility, and commercial vendors also have proprietary algorithms to ensure that medical models have the greatest positive impact. As a community grows, known areas for which there can be compromises in quality will likely be magnified by the sheer number of users. This will be compounded by the development and use of software or hardware that have not undergone rigorous vetting in the scientific and regulatory community.

Many of the concepts presented here lead the authors to believe that there exists a need for standardization across clinical sites using 3D printing technologies. Standardization would ensure the first steps towards upholding patient safety as paramount. It would also help to ensure that hospitals are following best practices from both the operational standpoint, and the FDA regulatory standpoint. There is also a large need for two sets of guidelines. The first is technical guidelines for models in Groups I, II, and III that will meet physician and patient needs. The second set are clinical guidelines that outline appropriateness of 3D printing for specific clinical scenarios, akin to those now produced by the American College of Radiology Appropriateness Criteria®.

## Conclusion

E plurbus unum (translation “out of many, one”) generally refers to the “melting pot” of many individuals, for example from different ancestries, religions, and races. 3D printing in medicine will emerge as a universal tool, a melting pot for the delivery of medical care that integrates medical information from volumetric imaging devices. The relatively small number of users will expand. More widespread enthusiasm will ultimately be fueled by reimbursement. During this transition, we are obligated to pay particular attention to quality medical modeling, and we will rely on the FDA to ensure safety and efficacy.

Very close collaboration between academics, industry, and the FDA is paramount to ensure safety for those U.S. patients whose quality of life will be improved by 3D printing. This article is designed to foster that collaboration and to encourage dialogue. Similarly, professionals engaged in 3D printing must maintain a high level of training and competence, and we believe that they should use FDA cleared software and hardware when appropriate. Education and clinical practice that focus on alternate, non-cleared software and hardware should be discouraged. For models designed to identically emulate patient anatomy, all hardware used to generate the image and post-process the relevant anatomy should be cleared by the FDA, while those steps related to the fabrication alone should be done with care, but should ultimately be monitored by the medical team caring for the patient in the Practice of Medicine. Patients for whom anatomy is altered significantly require use of software/hardware for that intended use which is cleared as a medical device. 3D printing has emerged as revolutionary paradigm shift for helping surgeons and interventionalists perform procedures in a more informed way, but for these patients and keeping safety at the forefront, all steps should be considered under the auspices of the FDA.
